# Consequences of the lack of clinical forensic medicine in emergency departments

**DOI:** 10.1007/s00414-023-02973-8

**Published:** 2023-02-20

**Authors:** Cristina Cattaneo, Stefano Tambuzzi, Stefano De Vecchi, Lidia Maggioni, Giorgio Costantino

**Affiliations:** 1 Istituto di Medicina Legale, Dipartimento di Scienze Biomediche per la Salute, Università degli Studi di Milano, via Luigi Mangiagalli, 37, 20133 Milan, Italy; 2 Fondazione IRCCS Ca’ Granda, Ospedale Maggiore Policlinico, Milan, Italy

**Keywords:** Clinical forensic medicine, Violence by others, Emergency departments, Forensics in hospitals

## Abstract

Most victims of physical violence sooner or later will access a hospital or medical cabinet because of that violence, and in particular emergency departments (EDs). This paper aims to analyze the performance of emergency ward clinicians in the forensic management of such victims by examining the activities carried out and the data reported. A total of 991 medical records were extrapolated from the database of the ED of the Policlinico of Milan in an average pre-pandemic 1-year activity. For each medical record, 16 parameters were analyzed in-depth including epidemiological data, information on the type of violent actions, injuries, and time between the infliction of the lesion and access to the ED. In the vast majority of cases, all the actions with medicolegal implications had been neglected by health professionals causing loss of data not only for the justice system but especially for correctly interpreting what happened and taking appropriate measures to protect the patient/victim. Hence, given that clinicians in EDs are busy with non-forensic clinical tasks (and rightly so), it should be ensured that there be specific forensic clinical personnel. However, it is crucial that when unfortunately there can be no forensic staff, at least the clinicians who work in the ED are properly trained to correctly apply essential medicolegal measures. Overall, timely and informed medical and forensic intervention is possible and necessary for the improvement and maintenance of the mental and physical health of victims of violence.

## Introduction

Interpersonal violence refers to violence between individuals and is divided into family and intimate partner violence and community violence, according to WHO [1]. Of course, this definition includes the most common types of violence, such as domestic violence, sexual violence, child abuse, and elder abuse. Interpersonal violence globally kills more than 1.6 millions of people every year, but the impact of non-fatal violence is also enormous and has serious long-term physical, psychological, economic, and social consequences [1]. Violence is quite common in today’s society, but in many cases it is a hidden phenomenon, leading to underestimation and inadequate resources to properly address the problem [1–3].

Despite the importance of properly recognizing and treating violence from a medical point of view, health professionals, who have a crucial role in the detection and prevention of violence, seem to be currently not yet prepared to treat this kind of “disease” appropriately. The few existing publications on this issue highlight the inadequate preparation of health professionals in hospitals in this sense. Recently, Vieira et al. [4], through a series of questionnaires, demonstrated that in Brazilian hospitals most of the doctors and nurses knew less than 50% of the procedures required for the proper documentation, collection, and preservation of forensic evidence [4]. Specialist centers for domestic, sexual, and child abuse exist; however, these are usually located in large cities and scarcely distributed in single regions and countries. For this reason, all medical centers should have personnel capable of detecting and responding to clinical signs of violence given that reading through medical science crime or abuse on a body is functional to preserving health and life. Hence, in emergency wards especially the documentation and correct interpretation of voluntary injuries upon the medical and/or surgical intervention are crucial to protect the patients’ health in its broadest sense; yet this does not seem to occur.

For this reason, we decided to carry out an examination of all the cases admitted to the emergency department (ED) of the Policlinico of Milan, one of the most important hospitals in Lombardy (North Italy), in a pre-pandemic 1-year activity, to assess how the cases labeled as “violence by others” were treated from a descriptive and diagnostic point of view. The aim was to analyze, for the first time, the performance of emergency room physicians in the management of victims of violence, evaluating the set of assessments carried out by physicians during the examination of patients, the activities performed, and, finally, the data actually reported in the patients’ medical records. Particular emphasis was placed on monitoring the quantity and quality of samples taken for forensic purposes. We therefore present the results and discuss their implications and critical aspects from a medicolegal and clinical perspective, presenting a flowchart for forensic precautions that we believe should be part of the cultural heritage of all clinical practitioners in EDs and more generally in hospitals.

## Materials and methods

The medical records of all patients who were admitted to the emergency department of the Policlinico Hospital of Milan in 2017 were reviewed. All the medical records that had been labeled by health professionals as “patient victim of violence by others” were extrapolated completely anonymously. This may have involved non-sexual physical assault as well as sexual assault: the study assessed only the ability of clinicians at the ED to describe, evaluate, and treat violence, regardless of the fact that some of these victims might later be referred to a specialized sexual violence center. Subsequently, the study of the enrolled cases was implemented by extracting a series of common data, which are relevant both in the clinical and forensic field. As a result, 16 parameters were analyzed for each selected medical record, including epidemiological data, information about the characteristics of the aggression, and the clinical exams performed, as well as the description of findings and sampling carried out, as listed below:

- General patient information: gender (male; female) and age (divided into subgroups of 10 years each: 0–9, 10–19, 20–29, 30–39, 40–49, 50–59, 60–69, 70–79, 80–89, 90–99, or not known). The division into age decades corresponded only to the need to divide the population into homogeneous age groups. In Italy, the age of majority is reached at 18 years of age, and children and adolescents up to this age are admitted to the pediatric ED, yet given that the databases between the pediatric ED and the adult one are merged, we kept the adolescent limit at 19 since adolescence is traditionally considered complete at 19 years of age approximately [5].
- Time of transport and arrival at the emergency department: elapsed time between event and patient arrival at the ED (< 2 h, 2–4 h, 4–6 h, 6–11h, 11–16 h, 16–24 h, 24–48 h, > 48h, or not known).
- Information on injuries: both type of trauma claimed by the patient and diagnosed by the clinician (blunt force trauma, sharp force trauma, firearm, thermal, electric, asphyxia, intoxication/poisoning, or not known); type of injuries diagnosed by the clinician based on their morphological features (e.g., bruises, abrasions, skin lacerations, fractures, dislocations, scars, etc., or not known); was the injury suffered by the patient described by the health care provider? (yes, no, or not known); if yes, which information had been reported? In detail: injury’s characteristics (number of lesions, size, color, and shape) and body location. In the case of multiple coexisting injuries in the same individual, we considered the best descriptive performance of the health professional for the purposes of the descriptive statistics of this study.
- Information collected and specimens sampled at the ED: any instrumental examinations performed to better interpret the injury (e.g., X-ray or computerized tomography); were any photographs of the injuries taken? (yes, no, or not known); was the victim’s clothing sampled (yes, no, or not known).
- Clinical examination result: was the patient admitted to a hospital ward? (yes, no, or not known); was the Judicial Authority involved? (yes, no, or not known); what was the patient’s prognosis? (no prognosis, 2 days, 3–5 days, 6–10 days, 11–20 days, > 20 days, reserved prognosis, or not known). Prognosis is the number of days the physician estimates it will take for the patient’s injuries to heal.

## Results

**Fig. 1:**
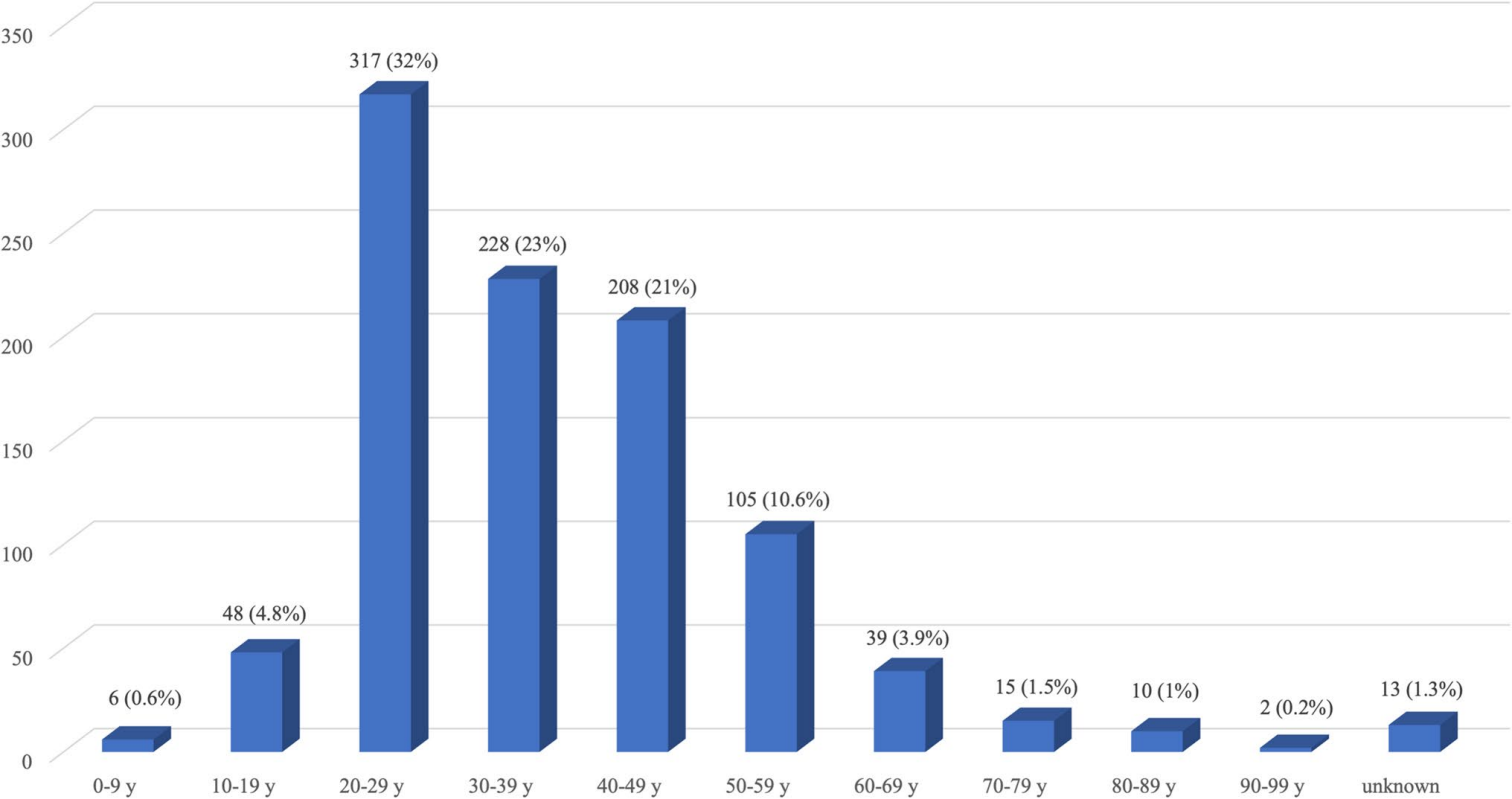
Schematic representation of age of the enrolled patients

There were a total of 80,934 hospitalizations at the ED of Policlinico of Milan in the year examined, of which 58,598 were registered at the adult ED and 22,336 at the pediatric ED. Out of the total admissions, 991 (1.22%) were patients that had been victim of violence configuring crimes against persons according to the Italian criminal code. In detail, in 761 cases (76.8%), it was general physical violence by others (i.e., assaults, fights, quarrels), and in the remaining 230 cases (23.2%), it was sexual violence. Overall, 519 (52.4%) victims referred to male, and 472 (47.6%) to female patients. The breakdown of the victims’ gender by the type of violence suffered showed that in the case of non-sexual physical violence, 470 were men (61.7%) and 291 were women (38.3%); in contrast, in the case of sexual violence, more than three-quarters of the victims were women (181, 78.7%). The number of admissions of patients at the ED varied according to the age group, as shown in Fig. 1. In a very small number of cases (13 cases, 1.3%), the victim’s age was unknown since the patient had left the ED before the clinical examination. However, it was found that among male victims of non-sexual physical violence, the age range was from 5 to 81 years, with an average age of 28.6 ± 17 years; among female victims, the age range was from 10 to 95 years, with an average age of 33.7 ± 15 years. The age of male victims of sexual violence ranged from 12 to 37 years, with an average age of 24.3 ± 6 years; for female victims, the age ranged from 4 to 68 years, with an average age of 30.5 ± 11 years. Globally, a total of 52 minors (under 18 years of age) suffered some type of physical violence (5.3% of 978 patients with known age), distributed as follows: for non-sexual violence, there were 18 (58%) males and 13 (42%) females; for sexual violence, there were 4 (19%) males and 17 (81%) females. Among adults, the total number of victims of physical violence was 926 (93.4% of all patients with known age), distributed as follows: for non-sexual violence, 448 (62%) men and 274 (38%) women; for sexual violence, 45 (22%) men and 159 (78%) women.

After analyzing the time of arrival of patients to the ED (which was reported in 618 cases, 62.4% out of the total), we observed that 337 (54.5%) victims arrived at the ED within 2 h after having suffered the violence. After that, 124 (20%) victims arrived between 2 and 4 h, 43 (7%) between 4 and 6 h, 33 (5.4%) between 6 and 11 h, 28 (4.5%) between 11 and 16 h, and 16 (2.6%) between 16 and 24 h. Admissions were also registered after more than 24 h from the violence, in detail: 29 (4.7%) between 24 and 48 h and 8 (1.3%) after 48 h. Specific data broken down by the time of arrival at the ED, type of violence suffered (non-sexual and sexual), and age of victims are reported in Table 1. Unfortunately, in 373 cases (37.6% out of the total), involving both types of violence and both adults and minors, it was not possible to assess the elapsed time before the patient’s arrival at the ED, since the time of the violence by others had not been reported in medical records.

**Table 1:** Summary table of the breakdown by the time of arrival at the ED, type of violence suffered, and age of victims

Time of arrival at the ED (h)	Total numbers (%)	Adults (> 18 years)		Minors (< 18 years)	
		Non-sexual (%)	Sexual (%)	Non-sexual (%)	Sexual (%)
Within 2	337 (54.5)	318 (67)	12 (11.2)	7 (31.9)	
2–4	124 (20)	94 (19.8)	26 (24)	4 (18.2)	
4–6	43 (7)	29 (6.1)	12 (11.2)	2 (9.1)	
6–11	33 (5.4)	16 (3.4)	13 (12)	3 (13.6)	1 (7.7)
11–16	28 (4.5)	10 (2.1)	14 (13)	1 (4.5)	3 (23)
16–24	16 (2.6)	3 (0.6)	9 (8.3)	2 (9.1)	2 (15.4)
24–48	29 (4.7)	4 (0.8)	17 (15.7)	3 (13.6)	5 (38.5)
> 48	8 (1.3)	1 (0.2)	5 (4.6)		2 (15.4)
Total	618	475	108	22	13
Not reported	373 (37.6)				

**Fig. 2:**
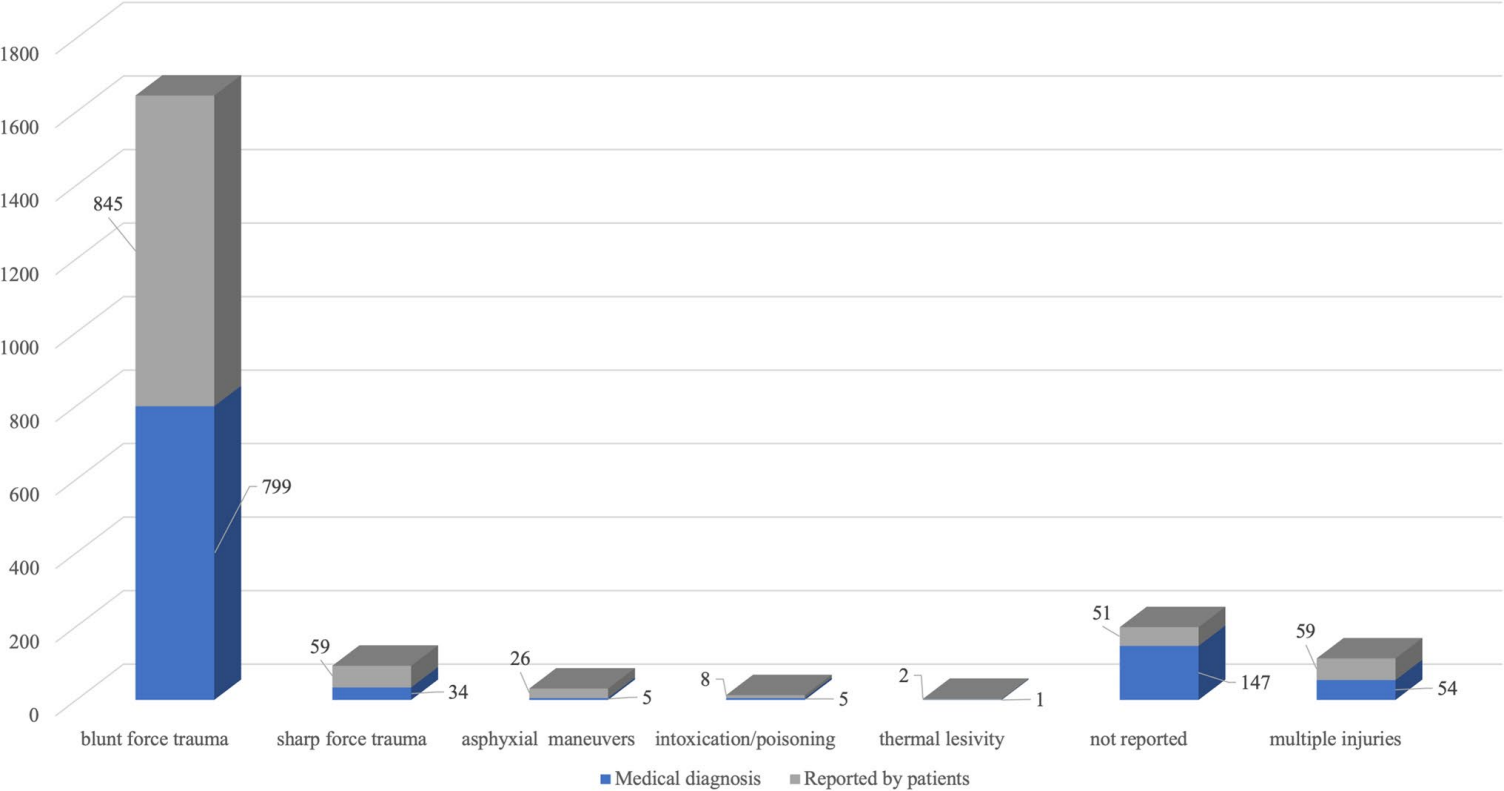
Schematic comparison between the claimed typology of injuries by patients and the diagnoses made by clinicians

Considering the reported type of trauma by the patients, out of the total we observed that 845 (85.3%) claimed to have suffered from blunt force trauma, 59 (6%) from sharp force trauma, 26 (2.6%) from asphyxial maneuvers (ligature or manual strangulation of the neck), 8 (0.8%) from intoxication/poisoning, and 2 (0.2%) from thermal trauma. In 51 cases (5.1%), the reported type of trauma by the patients had not been noted in medical records (in 13 cases, patients had left the ED before the clinical evaluation). Finally, out of the total, 59 patients claimed to have been victim of multiple types of injury (Fig. 2) and the most common associations were blunt force trauma and asphyxial maneuvers, followed by blunt force trauma and sharp force trauma. Following the medical examination, the diagnoses made by clinicians were as follows: blunt force trauma in 799 patients (80.6%), sharp force trauma in 34 patients (3.4%), asphyxial maneuvers in 5 patients (0.5%), intoxication/poisoning in 5 patients (0.5%), and thermal trauma in 1 patient (0.1%) (Fig. 2). In 147 cases (14.9%), the diagnosis was absent in medical records. Specific data broken down by the reported type of trauma by the patients and diagnosed by the clinicians, type of violence suffered (non-sexual and sexual), and age of victims are reported in Table 2. For the sake of completeness, it should be mentioned that blood samples are taken from all victims who claim to have suffered sexual violence in order to perform toxicological screening; however, this is not done routinely for victims of non-sexual physical violence.

**Table 2:** Summary table of the breakdown by the reported type of trauma by the patients and diagnosed by the clinicians, type of violence occurred (non-sexual and sexual), and age of victims

	Reported by patients					Medical diagnosis				
	Tot. (%)	Adults (> 18 years)		Minors (< 18 years)		Tot. (%)	Adults (> 18 years)		Minors (< 18 years)	
		Non-sexual (%)	Sexual (%)	Non-sexual (%)	Sexual (%)		Non-sexual (%)	Sexual (%)	Non-sexual (%)	Sexual (%)
Blunt force	845 (90)	659 (94)	156 (81.2)	17 (60.6)	13 (72.3)	799 (94.7)	649 (96.4)	125 (89.3)	14 (77.8)	11 (84.6)
Sharp force	59 (6.3)	38 (5.4)	16 (8.4)	5 (17.9)		34 (4)	23 (3.5)	9 (6.5)	2 (11.2)	
Asphyxia	26 (2.7)	4 (0.5)	13 (6.8)	5 (17.9)	4 (22.2)	5 (0.6)	1 (0.1)	2 (1.4)	1 (5.5)	1 (7.7)
Intoxication	8 (0.8)		7 (3.6)		1 (5.5)	5 (0.6)		4 (2.8)		1 (7.7)
Thermal	2 (0.2)	1 (0.1)		1 (3.6)		1 (0.1)			1 (5.5)	
Total	940	702	192	28	18	844	673	140	18	13
Not reported	51 (5.1% out of the total)					147 (14.9% out of the total)				

Focusing specifically on injury description and diagnosis made by clinicians, we observed that bruises had been reported in 519 cases, abrasions in 246 cases, skin lacerations in 211 cases, and bone fractures in 112 cases.

**Fig. 3:**
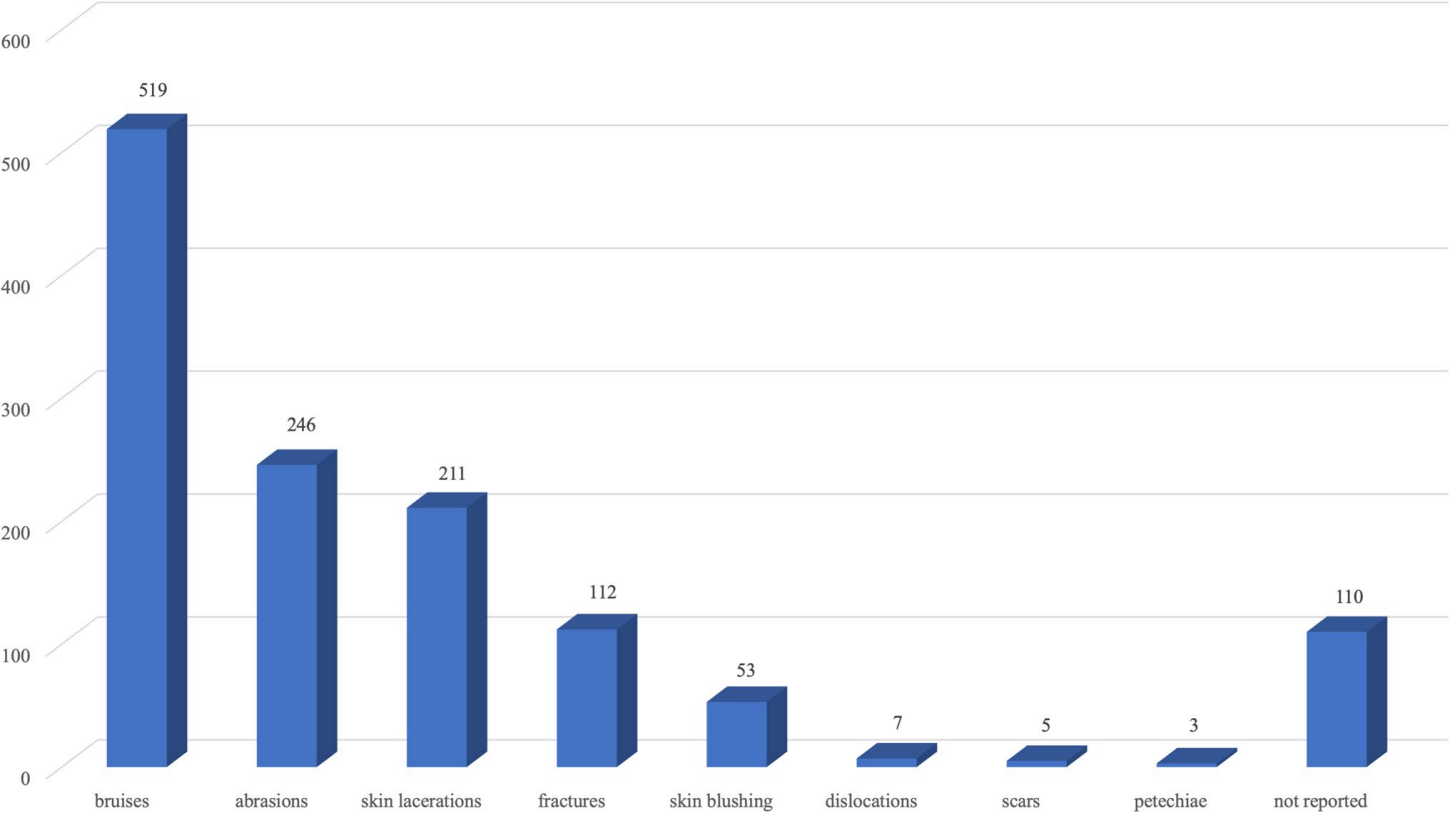
Schematic representation of the injuries observed by clinicians on the patients

Bruising and skin abrasions occurred in all types of patients, in 296 cases (57%) and 115 cases (46.8%), respectively, in adult victims of non-sexual violence, in 169 cases (32.6%) and 74 cases (30%) in adult victims of sexual violence, in 35 cases (6.8%) and 33 cases (13.5%) in minor victims of non-sexual violence, and in 19 cases (3.6%) and 24 cases (9.7%) in minor victims of sexual violence, respectively. In contrast, skin lacerations and bone fractures were predominantly found in young adult victims of non-sexual physical violence in 137 cases (65%) and 74 cases (66%), respectively. Skin reddening, dislocations, scars, and petechiae were reported way less frequently in all victim types. In 110 cases of the total clinically examined victims (11%), the type of reported injury, or lack of objective lesions, had not been noted on the medical records (Fig. 3). Of course, different types of injuries could coexist in the same patients overall, and the most common associations were bruises and abrasions, as well as bruises and skin lacerations.

We also investigated how in-depth the injuries had been described on medical reports by clinicians. Of the total number of patients who underwent clinical evaluation, in 933 cases (95.3%) at least one of the description parameters taken into consideration was reported, whereas in 44 cases (4.5%) there was no injury description. In 1 case (0.2%), it was not possible to ascertain whether the description had been made or not. The size of the injury was reported in 95 cases (10.2%), the color in 59 cases (6.3%), the shape in 61 cases (6.5%), and the location in 718 cases (77%). The total number of lesions was noted in 76 cases. Specific data broken down by the type of violence suffered (non-sexual and sexual) and age of victims are reported in Table 3. In the case of multiple coexisting injuries in the same individual, the best descriptive performance was considered.

**Table 3:** Summary table of the breakdown by the descriptive performance of injuries made by clinicians, type of violence occurred (non-sexual and sexual), and age of victims

	Tot. (%)	Adults (> 18 years)		Minors (< 18 years)	
		Non-sexual (%)	Sexual (%)	Non-sexual (%)	Sexual (%)
Size	95 (10.2)	52 (8.6)	22 (9.5)	8 (16.7)	13 (24.5)
Color	59 (6.3)	19 (3.2)	25 (10.8)	6 (12.5)	9 (17)
Shape	61 (6.5)	21 (3.5)	17 (7.4)	9 (18.8)	14 (26.4)
Location	718 (77)	509 (84.7)	167 (72.3)	25 (52)	17 (32)
At least one of reported parameters	933	601	231	48	53
Total number of lesion	76	27	24	14	11
Absent/not assessable	58 (5.8)				

Body location of the reported injuries was the following: head in 235 patients (32.8%), face in 384 patients (53.5%), neck in 19 cases (2.6%), chest in 88 patients (12.3%), abdomen in 38 patients (5.3%), upper limbs in 118 patients (26.1%), lower limbs in 115 patients (16.1%), back in 47 patients (6.5%), genitalia in 10 patients (1.3%), and buttocks in 22 patients (3.0%). Details are shown in Table 4. We note that the percentages given refer to the totality of injuries for which anatomical location was reported in the description, since in some cases the same individual had multiple injuries in different anatomical areas. However, of the total 978 subjects who underwent clinical evaluation, only 718 had at least one lesion for which the affected anatomic site was noted; in 260 cases (26.6%), this information was missing.

**Table 4:** Summary table of the breakdown by the anatomical sites affected by injuries, type of violence occurred (non-sexual and sexual), and age of victims

	Tot. (%)	Adults (> 18 years)		Minors (< 18 years)	
		Non-sexual (%)	Sexual (%)	Non-sexual (%)	Sexual (%)
Head	235 (32.8)	123 (52.3)	67 (28.5)	29 (12.4)	16 (6.8)
Face	384 (53.5)	211 (55)	124 (32.3)	35 (9.1)	14 (3.6)
Neck	19 (2.6)	4 (21)	7 (36.8)	5 (26.4)	3 (15.8)
Chest	88 (12.3)	58 (66)	13 (14.7)	11 (12.5)	6 (6.8)
Abdomen	38 (5.3)	12 (31.6)	15 (39.5)	4 (10.5)	7 (18.4)
Upper limbs	118 (26.1)	59 (50)	31 (26.3)	17 (14.4)	11 (9.3)
Lower limbs	115 (16.1)	38 (33)	43 (37.5)	15 (13)	19 (16.5)
Back	47 (6.5)	24 (51)	11 (23.4)	7 (14.9)	5 (10.7)
Genitalia	10 (1.3)	1 (10)	6 (60)	0	3 (30)
Buttocks	22 (3)	3 (13.6)	5 (22.7)	8 (36.3)	6 (27.4)
Absent			260 (26.6)		

As for tests that had been prescribed by clinicians, X-ray examinations were performed in 416 cases (42.5%) and computed tomography scans in 357 cases (36.5%). These instrumental examinations were predominantly performed in adult victims of non-sexual physical violence in 305 cases (73.3%) and 259 cases (72.6%), respectively, followed by adult victims of sexual violence in 83 cases (20%) and 93 cases (26%), minor victims of non-sexual violence in 20 cases (4.7%) and 5 cases (1.4%), and minor victims of sexual violence (X-ray in 8 cases, 2%). Ultrasound and echo-Doppler examinations were also reported in a few cases (exclusively in adults). No instrumental examinations were performed in 198 cases (20.2%).

**Fig. 4:**
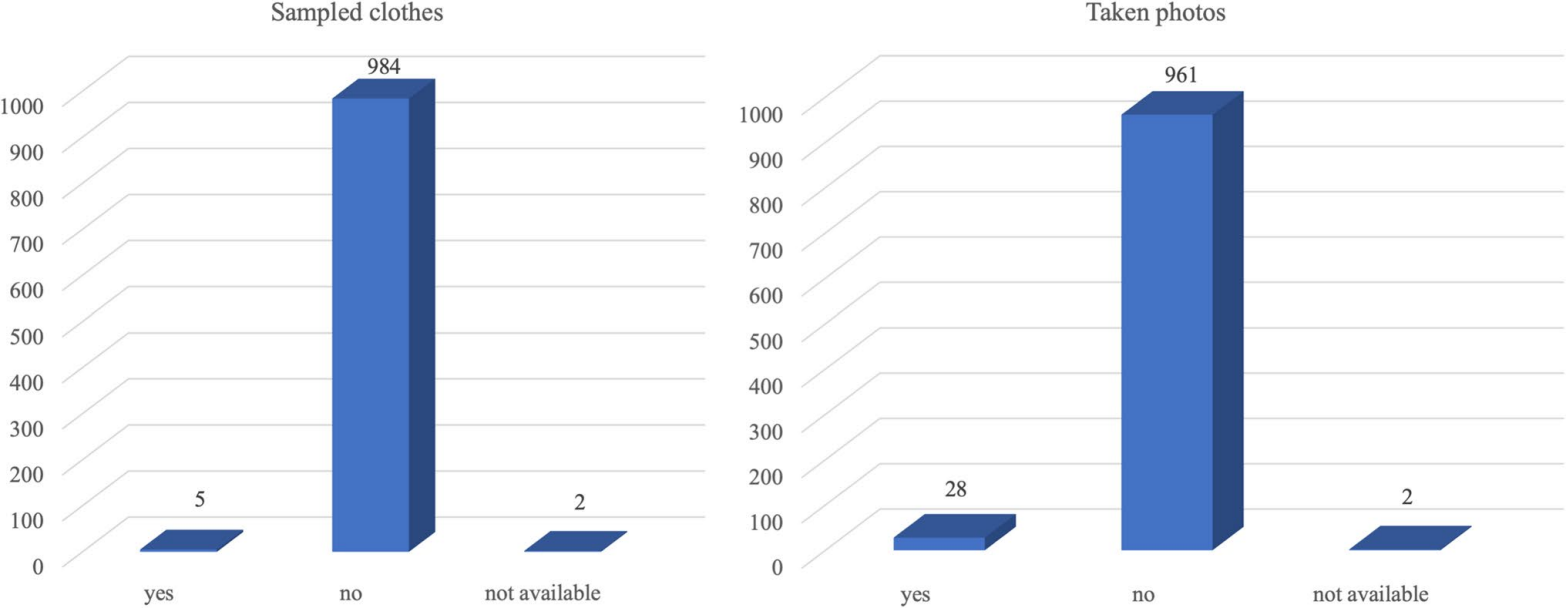
Schematic representation of the frequency of sampling clothes (on the left) and taking photos (on the right)

We observed that in 984 cases (99.3%) no clothes belonging to the victim or the aggressor were kept; only in 5 cases (0.5%), all relating to non-sexual physical violence in adults, some clothes were seized, and in the remaining 2 cases (0.2%), this information was not available (Fig. 4). Furthermore, in 961 cases (97.0%), no photos of the reported injuries were taken by the health professionals; in 28 cases (2.8%), photos were taken; and in the remaining 2 cases (0.2%), this information was not available (Fig. 4). Specifically, photographs of injuries were taken in 17 cases (60.7) of adult victims of non-sexual violence, in 5 cases (17.8%) of adult victims of sexual violence, in 2 cases (7.1%) of minor victims of non-sexual violence, and in 4 cases (14.4) of minor victims of sexual violence. The report to the judicial authority was made in 667 cases (67.3%) when the crimes could be prosecuted ex officio and there was an obligation to report.

Finally, after admission at the ED, 59 patients (6%) were hospitalized, while in the remaining 919 cases (94%), they were discharged (it is recalled that 13 patients left before the medical examination). For 225 patients (23%), there was no prognosis. It was 2 days in 20 patients (2%), 3–5 days in 162 patients (16.6%), 6–10 days in 363 patients (37.2%), 11–20 days in 108 patients (11%), and > 20 days in 92 patients (9.4%); it was reserved in 8 patients (0.8%). Adult victims of non-sexual physical violence reported a longer prognosis, 7 days on average.

## Discussion

Victims of violence very often visit hospital emergency wards to receive medical assistance. This is a fact that should induce the implementation of thorough medicolegal and forensic procedures for the appropriate treatment of these patients and for prevention. This is especially true given that violence is a pervasive phenomenon in today’s society. However, recent findings in the literature clearly show that emergency clinicians do not exercise due diligence in documenting, collecting, and preserving forensic evidence [4]. This is an excellent missed opportunity, however, as a patient’s entry into the ED immediately after a violent crime or even suspicious trauma would be the best time to properly diagnose and intercept violence in order to safeguard the health and life not only of that patient but of society, and provide preventive measures. Indeed, the forensic examinations that can be performed at the ED are several (descriptions, photographs, interpretations, and collection of biological specimens) and, by their nature, require little or no delay as the injured biological substrate of a living individual is subject to contamination or change until final recovery [6]. Therefore, timely examinations usually provide more accurate information than delayed assessments and may also prove to be critical in the differential diagnosis between an accidental and a non-accidental injury. These are considerations that may seem trivial, but data from the literature seems to indicate that these seemingly familiar concepts are not successfully applied in daily practice in the clinical settings [4]. Perhaps often not considered is that a patient who has suffered violence may also face legal proceedings at the end of the clinical process. Therefore, if the patient is injured, early implementation of forensic measures could allow for the collection of evidence necessary for future legal consequences, as well as taking measures to protect that particular individual. Ultimately, this would be the best way to protect the health of the victim in the broadest sense.

Given the importance of forensic medical intervention in emergency wards, we decided to carry out an examination of all the cases admitted to the emergency department of the Policlinico of Milan, one of the most important hospitals in Lombardy (North Italy), in a standard (pre-COVID) 1-year activity, to assess how the cases labeled as “violence by others” were dealt with from a descriptive and diagnostic point of view. Particular emphasis was placed on assessing the implementation of medicolegal measures by emergency clinicians in the management of patients who arrive classified as victims of violence. A total of 991 medical records were examined.

In our experience, slightly less than 80% of patients were victims of general physical violence by others, such as assaults and fights. The remaining 20% approximately, mostly composed of women, was victim of sexual violence. Overall, there was no significant difference between male and female gender, but the breakdown of results showed that more men were victims of non-sexual physical violence and more women were victims of sexual violence.

This was true for both minors (under 18 years in Italy) and adults. Overall, the age group most frequently affected by violence was between 19 and 49 years, and the average age for both types of violence (non-sexual and sexual) was around 30 years for both men and women. It is noteworthy that the average age of male victims of sexual violence was lower than that of female victims. This is consistent with data from the literature showing that older women are still at risk of becoming victims of sexual violence [7]. On the whole, all these findings are in line with the data provided by ISTAT (Italian National Statistics Institute) about the victims of physical and sexual violence [8]. Indeed, younger subjects may be more frequently victim of violence, since they are more socially active and prone to alcohol [9, 10] and illicit drug [11] assumption. Such substances may often lead to a violent behavior toward other individuals [12], triggering violence based on discriminating factors such as disability, ethnicity, religion, and sexuality [13]. Nevertheless, this information cannot be considered a representation of violence and victims in the city as many cases of more subtle violence may have been missed or misdiagnosed by clinicians and therefore not classified as violence upon arrival and hence not included in this study.

Through the careful analysis of the events that occurred, it was observed that 54% of patients arrived at the ED within 2 h from the event, and 27% within 2 and 6 h. This means very quick access for most patients and confirms how effective the adoption of medicolegal measures could be in evaluating injuries. However, a significant number of patients (20% approximately) went to the ED even after 6 h, which confirms that the request for help after violence can also be delayed in time [6], especially in cases of sexual violence, where the percentage rises to almost 60% for adults and 100% for minors. This is not surprising given the intimate nature of the violence experienced and the possible inner conflict when seeking help or telling parents what happened. Of great concern, however, was that in approximately 37% of cases (373 patients), the timing of the violence was not reported in the medical records.

In the vast majority of cases, patients reported being victims of blunt force trauma (85% approximately), regardless of the type of violence (non-sexual or sexual) and age. It was followed by sharp force trauma in all victims, with the only exception of minor victims of sexual violence, where asphyxial maneuvers took second place in terms of frequency. If in about 5% of the cases the injury claimed by the patient was not indicated in the medical record, the fact that in about 15% of the cases the medical diagnosis of the injury suffered by the patient was completely missing is even more worrying. To be realistic, we assume that any clinical physician is capable of diagnosing a blunt or sharp force trauma but perhaps asphyxial injuries are more challenging to interpret correctly as detecting signs such as petechiae may be trickier for inexperienced personnel. Consistently, the study found that the most commonly diagnosed injuries across all types of violence victims were bruises and abrasions. They were followed by skin lacerations and bone fractures that were most common among young adult victims of non-sexual physical violence. In contrast, all other types of injuries were reported in very few cases, especially petechiae in 3 cases only. In general, according to the literature [14], the head and face were the most commonly affected body sites (about 32% and 53%, respectively), followed by the upper limbs. In particular, the upper and lower limbs were most commonly affected in cases of sexual violence, both in adults and minors.

Since from a medicolegal point of view it is crucial to properly describe the injuries, we assessed if the emergency room clinicians noted all the relevant characteristics, such as size, color, shape, location, and number of lesions. The site of the lesion was quite often described (77% of cases), unlike all the other parameters, which were almost always absent or incomplete. Indeed, the injury size was reported only in 10.2% of cases, the color in 6.3% of cases, and the shape in 6.5% of cases. This deficiency in lesion description affected all types of patients, but the breakdown of the results showed that all the parameters of injury description were better reported in victims of sexual violence than in victims of non-sexual physical violence, with the sole exception of injury localization. This seems to indicate greater (perhaps unconscious) attention by clinicians when dealing with cases of sexual violence injuries. However, almost all of the reports assessed in this study were uninformative from a medicolegal perspective and of little relevance in a forensic setting. Indeed, a poor description does not allow at a later stage to retrospectively assess the injury severity, timing of onset, and evolution, possibly causing an underestimation and not allowing a correct assessment of the consistency between history and lesions suffered [11, 15, 16]. Furthermore behind a poor description may lie poor interpretation and consequently a lesser inclination to a more thorough investigation of wound nature and timing.

In almost 80% of cases, emergency physicians requested imaging tests, and almost exclusively X-rays and computed tomography scans, primarily for adult victims of non-sexual physical violence. For other victims, far fewer instrumental examinations were performed. However, this approach not only allows for a more comprehensive clinical examination that ensures a higher level of care, but may also provide elements that can be used later from a medicolegal perspective, such as detecting signs of physical abuse (in the case of multiple bone calluses) or assessing the correspondence between the reported bone fracture and the type of trauma that occurred [17]. Adult victims of non-sexual physical violence reported a longer prognosis, 7 days on average, and this inevitably also draws attention to the economic and social costs of violence.

However, in addition to the poor descriptions of injuries, we also observed more critical issues in other procedures that have a purely forensic purpose, but should never be overlooked [18–20]. Photographs of injuries were taken only in 28 patients (2.8% of cases), with very low numbers in all victim types. However, the acquisition of photographs is of fundamental importance since evidence rapidly changes [21]. Indeed, since photos can document the initial aspect of lesions, they may have a crucial role in court. Also, the preservation of clothing belonging to the victim can be pivotal for investigative purposes, since they may be subjected to forensic genetic examination to search for perpetrator’s biological traces. However, only in 5 patients (0.5%), all relating to non-sexual physical violence in adults, was the victim’s clothing kept. Again, these are seemingly commonplace considerations, but reality shows that while these concepts are widely known, their practical application is completely neglected.

Of course, the primary purpose of healthcare professionals in the emergency department is to identify life-threatening conditions and address them. However, when this task has been accomplished, or if the conditions were not life-threatening, care for the patient’s health in its broadest sense should also take over. This also involves understanding whether that patient has been a victim of violence and how to protect him or her. Therefore, the role of physicians, surgeons, or nurses should consist also in assessing if that injury may have been intentional or not. To do that, it is critical to observe, describe, document, test, and then verify nature, timing, and mode of production of injuries. It is expected that a patient arriving at the ED with chest pain will have at least a thorough medical history, blood tests, and electrocardiogram (ECG) [22]. The same approach should apply to a child with a broken arm, for example, which has been attributed to an accidental fall. Tests, X-rays, or even chest scans or MRIs, and whatever else is necessary, should be performed to see if type and pattern of injury as well as timing are consistent with the history. If violence is not hypothesized and maltreatment not suspected, nothing will be done to protect the patient’s health and life [23]. Therefore, violence should begin to be considered on the same level as any other disease [24] and, if not diagnosed and addressed, can lead to health deterioration or even have lethal outcomes, as in the case of feminicides [25, 26].

**Fig. 5:**
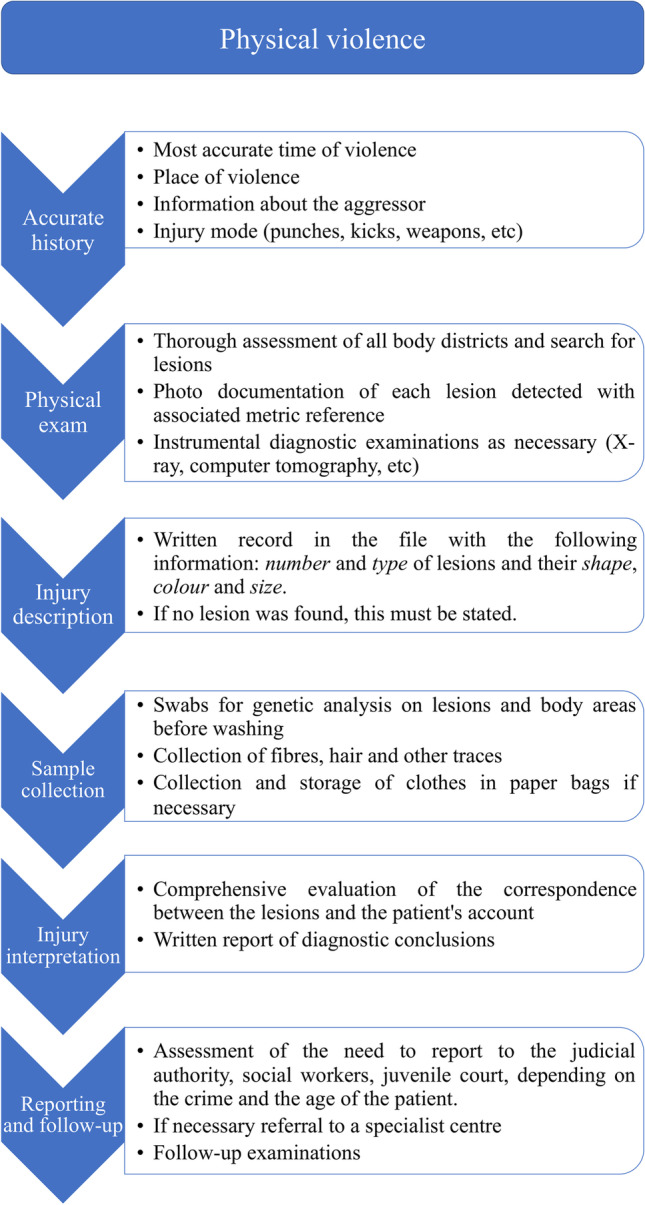
Schematic flowchart of the essential medicolegal measures

Whether this should be the role of all first responders or, more reasonably, whether forensic physicians should be present for these specific tasks in all hospitals is a political decision. But there is no doubt that this clinical service should be professionally dispensed in all hospitals, as it has been introduced in France through reforms and investments [27]. Therefore, it is crucial that even in hospitals where there is no forensic staff, at least the clinicians who work in the emergency department are properly trained and prepared. Since they are rightly primarily concerned with the purely clinical aspects, a good compromise might be to adopt and correctly apply some essential medicolegal measures that can be applied to all the cases of violence in general, which have been briefly summarized in the flowchart in Fig. 5. Overall, it is, first and foremost, a health and public health issue. The steps taken to correctly interpret and then diagnose violence are critical to protect health and life through medical intervention. This is the main reason why it is crucial that the culture of diagnosing violence is ingrained in all physicians, just like cancer and heart disease [12, 28, 29]. A good start would be to equip emergency rooms with a kit containing an assigned camera for this purpose, and a metric reference to measure lesions and to include in the photographs, as well as swabs and vials. This should be flanked by the introduction of courses in which all these aspects can be dealt with and emergency room clinicians (both physicians and nurses as first responders) made aware of the importance of not neglecting measures and precautions that might seem exclusively of forensic interest. While this may seem directly interpreted as a service to justice (and indeed it is, which is why diatribes sometimes arise as to whether the justice system or the National Health System should pay for these expenses), in the end, it is always related to the mission of protecting health. Moreover, the collection of data and evidence is closely related to medical acts, such as swabbing genitals or other anatomical areas, taking intimate and anatomically clear photographs, describing a bruise appropriately, and collecting traces from a wound [6, 30–32]. By and large, proper training would provide health professionals with those indispensable but invaluable skills that would enable them to better deal with victims of violence. Moreover, since EDs are privileged observers of the impact of violence on the population, a better capacity to detect and intercept this phenomenon could have enormous consequences on the epidemiology of violence and prevention especially in the most fragile population groups such as children and elderly people. Of course, the enormous number of submerged cases of violence could also decrease.

In this context, it is very likely that there were patients in our study who were victims of violence and were not recognized as such by the treating physicians. However, this should be considered a limitation of the current organization at higher levels rather than a limitation of the study. Indeed, we focused on assessing the performance of emergency clinicians in medicolegal interventions when dealing with claimed cases of violence. The reported numbers, besides describing a cross-section of the phenomenon of violence in a society over the course of 1 year, have brought to light the current critical problems we have discussed. The issues do not end there, of course, because it would be necessary, for example, for the forensic samples taken to actually be stored and analyzed, which is not happening [33]. However, the introduction of forensic experts or at least skills into the EDs would be a major step forward. To date, this lack may be the equivalent of not having someone to take care of cardiological or neurological issues given the high frequency of violence. Of course, this does apply not only to Italy but also to other countries. In our opinion, given the large quantity of cases in EDs, and hence the fact that clinicians in EDs are busy with non-forensic clinical tasks (and rightly so), it should be ensured that there is specific forensic clinical personnel [34, 35].

## Conclusion

The correct fulfillment of a medicolegal protocol during the medical examination in the emergency rooms, at the first contact with a patient victim of violence, is essential not only for the correct future legal development but especially to guarantee the patient’s overall health. Lack of detailed history, descriptions, and photographs as well as of the sampling of evidence is probably due to the lack of time, resources, and specialized personnel in the emergency ward. Currently, proper medical and forensic interventions are necessary for the improvement and maintenance of the mental and physical health of the victims of violence.

## Data Availability

All the data have been reported in the manuscript.
